# Rare coding variant architecture and gene discovery from 130,000 sequenced cases of atrial fibrillation

**DOI:** 10.21203/rs.3.rs-9191644/v1

**Published:** 2026-05-04

**Authors:** Sean J Jurgens, Nobuyuki Enzan, Ian R Dinsmore, Seung Hoan Choi, Jon Luo, Alex Lipov, Cassandra Hartle, Xin Wang, Nicholas A Marston, Lu-Chen Weng, Giorgio EM Melloni, Brandon Chalazan, Michael P Gray, James P Pirruccello, Annette Diaz, Mark D Chaffin, Aylin Ornelas-Loredo, Owen Tang, Faisal A Darbar, Shinwan Kany, Yining Chen, Aenne S von Falkenhausen, Alanna C Morrison, Andrea Natale, Arnljot Tveit, Bastiaan Geelhoed, Brian Cade, David R Van Wagoner, Doreen Haase, Elsayed Z Soliman, Giovanni E Davogustto, Hugh Calkins, Jeffrey L Anderson, Jennifer A Brody, John Barnard, John E Hokanson, Jonathan D Smith, Joshua C Bis, Kendra Young, Linda SB Johnson, Leann Long, Lorenz Risch, Lorne J Gula, Lydia Coulter Kwee, Michael Kühne, Michael Preuss, Namrata Gupta, Navid A Nafissi, Nicholas L Smith, Peter M Nilsson, Pim van der Harst, Quinn S Wells, Renae L Judy, Renate B Schnabel, Renee Johnson, Roelof AJ Smit, Stacey Gabriel, Stacey Knight, Tetsushi Furukawa, Yuan-I Min, Zachary T Yoneda, Zachary WM Laksman, Alvaro Alonso, Bruce M Psaty, Christine M Albert, Dan E Arking, Dan M Roden, Daniel I Chasman, Daniel J Rader, David Conen, David D McManus, Diane Fatkin, Eric Boerwinkle, Gregory M Marcus, Ingrid E Christophersen, J. Gustav Smith, Jason D Roberts, Laura M Raffield, M. Benjamin Shoemaker, Michael H Cho, Michael J Cutler, Mina K Chung, Morten S Olesen, Moritz F Sinner, Nona Sotoodehnia, Paulus Kirchhof, Ruth JF Loos, Saman Nazarian, Sanghamitra Mohanty, Scott M Damrauer, Stefan Kaab, Susan R Heckbert, Susan Redline, Svati H Shah, Toshihiro Tanaka, Yusuke Ebana, Regeneron Genetics Center, NHLBI Trans-Omics for Precision Medicine (TOPMed) Consortium, Steven A Lubitz, Kathryn L Lunetta, Emelia J Benjamin, Michiel Rienstra, Gemma A Figtree, Dawood Darbar, Connie R Bezzina, Christian T Ruff, Marc S Sabatine, Tooraj Mirshahi, Patrick T Ellinor

**Affiliations:** 1 Cardiovascular Disease Initiative, Broad Institute of MIT and Harvard, Cambridge, MA, USA; 2 Department of Experimental Cardiology, Amsterdam Cardiovascular Sciences, Heart Failure & Arrhythmias, Amsterdam UMC location University of Amsterdam, Amsterdam, Netherlands; 3 Department of Molecular and Functional Genomics, Geisinger Health System, Danville, PA, USA; 4 Department of Biostatistics, Boston University School of Public Health, Boston, MA, USA; 5 Wellcome Sanger Institute, Cambridge University, Cambridge, UK; 6 TIMI Study Group, Boston, MA, USA; 7 Division of Cardiovascular Medicine, Brigham and Women’s Hospital, Harvard Medical School, Boston, MA, USA; 8 Cardiovascular Research Center, Massachusetts General Hospital, Harvard Medical School, Boston, MA, USA; 9 Department of Pediatrics, Division of Genetics, Genomics, and Metabolism, Lurie Children’s Hospital of Chicago, Chicago, IL, USA; 10 Department of Pharmacology, Northwestern University, Chicago, IL, USA; 11 Sydney Medical School, Faculty of Medicine and Health, University of Sydney, Sydney; 12 Cardiovascular Discovery Group, Kolling Institute of Medical Research, St Leonards, New South Wales, Australia; 13 Division of Cardiology, University of California San Francisco, San Francisco, CA, USA; 14 College of Liberal Arts and Sciences, University of Illinois Chicago, Chicago, IL, USA; 15 Division of Cardiology, University of Illinois Chicago, Chicago, IL, USA; 16 Department of Medicine, University of Illinois at Chicago, Chicago, IL, USA; 17 Department of Cardiology, University Heart and Vascular Center Hamburg, Hamburg, Germany; 18 Partner site North, German Center for Cardiovascular Research (DZHK), Hamburg, Germany; 19 Department of Medicine I, University Hospital Munich, Ludwig-Maximilians-University, Munich, Germany; 20 Partner site Munich Heart Alliance, German Centre for Cardiovascular Research (DZHK), Munich, Germany; 21 Human Genetics Center, Department of Epidemiology, School of Public Health, The University of Texas Health Science Center at Houston, Houston, TX, USA; 22 Texas Cardiac Arrhythmia Institute, St David’s Medical Center, Austin, TX, USA; 23 Department of Biomedicine and Prevention, Division of Cardiology, University of Tor Vergata, Rome, Italy; 24 Department of Medical Research, Bærum Hospital, Vestre Viken Hospital Trust, Gjettum, Norway; 25 Department of Cardiology, University Medical Center Groningen, University of Groningen, Groningen, Netherlands; 26 Division of Sleep and Circadian Disorders, Departments of Medicine and Neurology, Brigham and Women’s Hospital, Boston, MA, USA; 27 Department of Cardiovascular & Metabolic Sciences, Cleveland Clinic, Cleveland, OH, USA; 28 Atrial Fibrillation NETwork (AFNET), Münster, Germany; 29 Department of Internal Medicine, Cardiology Section, Epidemiological Cardiology Research Center (EPICARE), Wake Forest School of Medicine, Winston-Salem, NC, USA; 30 Department of Medicine, Division of Cardiovascular Medicine, Vanderbilt University Medical Center, Nashville, TN, USA; 31 Department of Medicine, Cardiology, Johns Hopkins University School of Medicine, Baltimore, MD, USA; 32 Intermountain Heart Institute, Intermountain Medical Center, Murray, UT, USA; 33 Division of Cardiology, University of Utah, Salt Lake City, UT, USA; 34 Cardiovascular Health Research Unit, Department of Medicine, University of Washington, Seattle, WA, USA; 35 Departments of Quantitative Health Sciences, Cleveland Clinic, Cleveland, OH, USA; 36 Anschutz Medical Campus, Department of Epidemiology, University of Colorado, Aurora, CO, USA; 37 Department of Clinical Physiology, Department of Clinical Sciences, Skåne University Hospital and Lund University, Lund, Sweden; 38 Department of Biostatistics and Data Science, Wake Forest School of Medicine, Winston-Salem, NC, USA; 39 Institute of Laboratory Medicine, Faculty of Medical Sciences, Private University of the Principality of Liechtenstein, Triesen, Liechtenstein; 40 Center of Laboratory Medicine, University Institute of Clinical Chemistry, University of Bern, Bern, Switzerland; 41 Section of Cardiac Electrophysiology, Division of Cardiology, Department of Medicine, Western University, London, Ontario, Canada; 42 Duke Molecular Physiology Institute, Duke University School of Medicine, Durham, NC, USA; 43 Cardiology/Electrophysiology, University Hospital Basel, University of Basel, Basel, Switzerland; 44 The Charles Bronfman Institute for Personalized Medicine, Icahn School of Medicine at Mount Sinai, New York, NY, USA; 45 Program in Medical and Population Genetics, Broad Institute of MIT and Harvard, Cambridge, MA, USA; 46 Division of Cardiology, Department of Medicine, Duke University School of Medicine, Durham, NC, USA; 47 Department of Epidemiology, University of Washington, Seattle, WA, USA; 48 Kaiser Permanente Washington Health Research Institute, Seattle, WA, USA; 49 Department of Clinical Sciences, Clinical Research Center, Lund University, Malmö, Sweden; 50 Department of Internal Medicine, Skåne University Hospital, Malmö, Sweden; 51 Department of Cardiology, University Medical Center Utrecht, Utrecht, Netherlands; 52 Departments of Medicine, Pharmacology, and Biomedical Informatics, Vanderbilt University Medical Center, Nashville, TN, USA; 53 Department of Surgery, Perelman School of Medicine, University of Pennsylvania, Philadelphia, PA, USA; 54 Department of Surgery, Corporal Michael Crescenz VA Medical Center, Philadelphia, PA, USA; 55 Partner site Hamburg/Kiel/Lübeck, German Center for Cardiovascular Research (DZHK), Hamburg, Germany; 56 Molecular Cardiology Division, Victor Chang Cardiac Research Institute, Sydney, New South Wales, Australia; 57 Faculty of Medicine and Health, School of Clinical Medicine, UNSW Sydney, Sydney, New South Wales, Australia; 58 Department of Medicine, University of Utah, Salt Lake City, UT, USA; 59 Department of Bio-Informational Pharmacology, Medical Research Institute, Institute of Science Tokyo, Tokyo, Japan; 60 Department of Medicine, University of Mississippi Medical Center, Jackson, MS, USA; 61 Department of Medicine and the School of Biomedical Engineering, University of British Columbia, Vancouver, British Columbia, Canada; 62 Department of Epidemiology, Rollins School of Public Health, Emory University, Atlanta, GA, USA; 63 Department of Cardiology, Smidt Heart Institute, Cedars Sinai Medical Center, Los Angeles, CA, USA; 64 McKusick-Nathans Institute, Department of Genetic Medicine, Johns Hopkins University School of Medicine, Baltimore, MD, USA; 65 Divisions of Preventive Medicine and Genetics, Brigham and Women’s Hospital, Harvard Medical School, Boston, MA, USA; 66 Division of Cardiovascular Medicine, Department of Medicine, Perelman School of Medicine at the University of Pennsylvania, Philadelphia, PA, USA; 67 Population Health Research Institute, McMaster University, Hamilton, Ontario, Canada; 68 University of Massachusetts Chan Medical School Worcester, Worcester, MA, USA; 69 Cardiology Department, St. Vincent’s Hospital, Sydney, New South Wales, Australia; 70 Department of Medical Genetics, Oslo University Hospital, Oslo, Norway; 71 Department of Cardiology, Lund University Diabetes Center and Wallenberg Center for Molecular Medicine and Clinical Sciences, Lund University and Skåne University Hospital, Lund, Sweden; 72 The Wallenberg Laboratory/Department of Molecular and Clinical Medicine, Institute of Medicine, Science for Life Laboratory, Gothenburg University and the Department of Cardiology, Sahlgrenska University Hospital, Gothenburg, Sweden; 73 Department of Genetics, University of North Carolina at Chapel Hill, Chapel Hill, NC, USA; 74 Channing Division of Network Medicine, Department of Medicine, Brigham and Women’s Hospital, Boston, MA, USA; 75 Department of Cardiovascular Medicine, Cleveland Clinic, Cleveland, OH, USA; 76 Laboratory for Molecular Cardiology, Department of Cardiology, Centre for Cardiac, Vascular, Pulmonary and Infectious Diseases, Copenhagen University Hospital Rigshospitalet, Copenhagen, Denmark; 77 Department of Biomedical Sciences, Faculty of Medicine and Health Sciences, University of Copenhagen, Copenhagen, Denmark; 78 Novo Nordisk Foundation Center for Basic Metabolic Research, Faculty of Health and Medical Sciences, University of Copenhagen, Copenhagen, Denmark; 79 Division of Cardiovascular Medicine, Hospital of the University of Pennsylvania, Philadelphia, PA, USA; 80 Department of Surgery and Department of Genetics, Perelman School of Medicine, University of Pennsylvania, Philadelphia, PA, USA; 81 Cardiovascular Health Research Unit, Department of Epidemiology, University of Washington, Seattle, WA, USA; 82 Department of Human Genetics and Disease Diversity and BioResource Research Center, Institute of Science Tokyo Graduate School of Medical and Dental Sciences, Tokyo, Japan; 83 Life Science and Bioethics Research Center, Institute of Science Tokyo, Tokyo, Japan; 84 Regeneron Genetics Center, Tarrytown, NY, USA; 85 NHLBI Trans-Omics for Precision Medicine (TOPMed) Consortium; 86 Demoulas Center for Cardiac Arrhythmias, Massachusetts General Hospital, Boston, MA, USA; 87 NHLBI and Boston University’s Framingham Heart Study, Framingham, MA, USA; 88 Department of Medicine, Boston University Chobanian & Avedisian School of Medicine, Boston Medical Center, Boston, MA, USA; 89 Department of Cardiology, Royal North Shore Hospital, Sydney, New South Wales, Australia

## Abstract

Rare coding genetic variants may exert large effects on risk of common disease, yet their contribution to disease architecture and their utility in gene prioritization remain limited by inadequate sample sizes. Here, we performed a massive-scale rare variant association study (RVAS), analyzing over 1.1 million sequenced participants among which 130,000 had atrial fibrillation (AF). Through a multi-mask burden testing approach, we identified 15 genes significantly associated with AF through rare large-effect variation. Integrative analyses revealed strong convergence between genes implicated by rare and common variation, and highlighted instances where RVAS data may aid in GWAS prioritization. Nevertheless, several RVAS genes were not among GWAS loci (*FAM189A2*, *ACTC1*, *FNIP1*, *FBN1*), or were not nominated through contemporary GWAS prioritization (*KDM5B, ZFP36L2*). Finally, we observed that ultra-rare protein-disrupting variants - concentrated in a small number of large-effect size genes - explained at least 2% of AF susceptibility across European and African ancestry groups. These findings refine the genetic architecture of AF, while highlighting the value and cost of RVAS for genomic discovery in common disease.

## Introduction

Genome-wide association studies (GWAS) have extensively mapped the common variant genetic architecture across thousands of diseases and traits^1,2^. Although GWAS have revealed hundreds of loci associated with complex diseases, individual signals often confer small effect sizes, function through uncertain causal genes, and show unclear biological magnitude and translational relevance^3,4^. In contrast, rare variant association studies (RVAS) using sequencing data can directly implicate genes in which protein disruption has a large, interpretable impact on disease risk^5–7^. As such, RVAS have the potential to identify core disease genes and core biological mechanisms, inform on the contribution of rare variation to disease architecture, and aid in causal gene prioritization from GWAS^7,8^. Nevertheless, RVAS of common diseases have been constrained by the need for very large sample sizes and by substantial methodological heterogeneity across studies. As a result, the contribution of rare variants to disease architecture and heritability remains poorly characterized for most common diseases.

Atrial fibrillation (AF) - the most prevalent sustained arrhythmia worldwide - represents an ideal model to address these challenges. AF is a major cause of stroke, heart failure, and death, with a substantial but incompletely characterized genetic basis. Prior GWAS have identified over 300 loci associated with AF, confirming a substantial common variant contribution to its heritability^9^. In contrast, RVAS have so far identified only a handful of genes reaching exome-wide significance^10,11^, leaving the role of rare coding variation to AF risk and heritability largely unknown. Moreover, while prior work suggests that GWAS and RVAS converge on shared causal genes and pathways in phenome-wide approaches^7^, the limited power of existing RVAS has precluded a systematic assessment of such convergence in AF.

Here, we assembled sequencing data from almost a million individuals, including 123,106 with AF, creating the largest sequencing-based RVAS dataset for any common disease to date. Using a multi-mask burden testing framework across eight cohorts with sequencing data, we systematically map the rare coding variant architecture of AF. We identify 15 genes significantly associated with AF risk, quantify the proportion of heritability attributable to rare protein-disrupting variants, and demonstrate strong convergence between RVAS and GWAS signals for AF. These findings refine the genetic architecture of a major common disease, while providing a generalizable framework for rare variant discovery and integrative analyses across RVAS and GWAS.

## Results

### Rare variant burden testing in over 123,000 AF cases from 8 datasets

**Figure 1:**
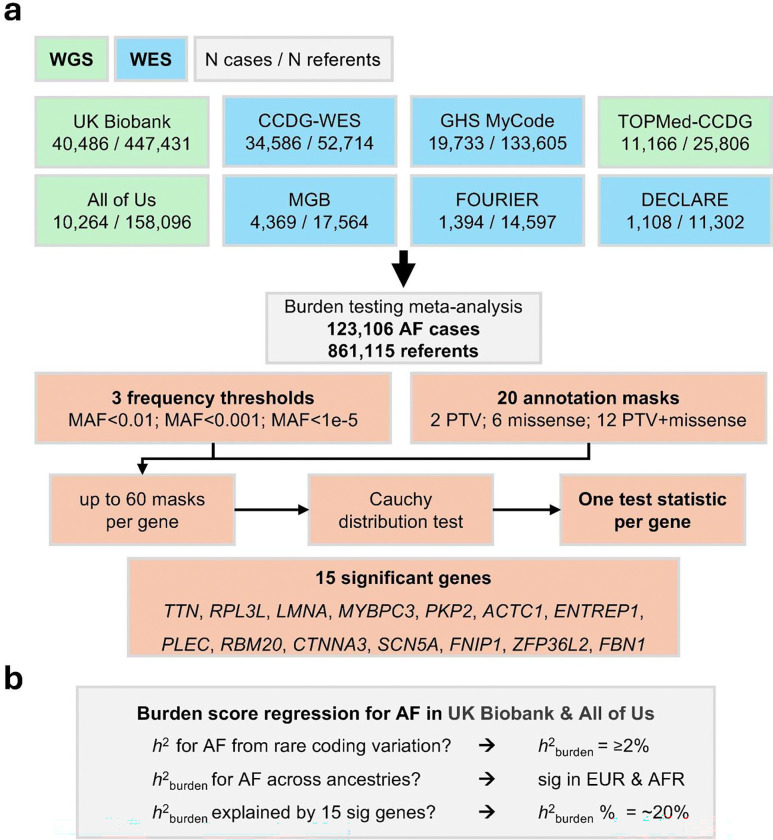
Study design. Part **a** is a flowchart describing the analytical workflow used to perform the gene-based rare variant association discovery analysis. As shown in the top of the figure, we collected data from 8 sequencing datasets, with either WGS (green) or WES (blue). Data from these 8 datasets, totalling 123,106 AF cases and 861,115 referents, was combined in a meta-analysis of gene-based burden tests. Below, the burden testing framework is shown in orange: Burden testing was performed using up to 3 frequency cutoffs and up to 20 annotation cutoffs, therefore allowing up to 60 different masks for every protein-coding gene; *P*-values were combined using the Cauchy distribution test to then produce one *P*-value per gene. Part **b** highlights the study analysis questions pertaining to genetic architecture of AF. We utilized burden score heritability regression, within WGS data from UK Biobank and All of Us, to estimate the proportion of AF susceptibility attributable to rare protein-disrupting variants. Abbreviations: WGS, whole-genome sequencing; WES, exome sequencing; N, number; h2, heritability; EUR, European genetic ancestry; AFR, African genetic ancestry.

We performed RVAS analyses for AF across 8 distinct datasets with whole-genome (WGS) or exome sequencing (WES) (Figure 1). The included datasets consisted of population-based and health-system cohorts (UK Biobank, Geisinger MyCode, All of Us Research Program, Massachusetts General Brigham Biobank), large case-control efforts assembled at least in part specifically to study AF (CCDG-WES, TOPMed-CCDG), and various clinical trials from the TIMI study group. Taken together, the datasets included 123,106 AF cases and 861,115 referents. Baseline characteristics for each dataset are provided in **Supplementary Table 1**. Further details, including ascertainment procedures, data processing, and quality-control, are presented in the **Supplementary Note** and **Supplementary Table 2.**

To perform our RVAS, we developed an extensive burden testing pipeline that was harmonized and applied across each of the datasets, followed by a meta-analysis (Figure 1 and Methods). In our burden testing approach, for any given protein-coding gene, we analyzed up to 60 different rare variant masks, in which we pooled rare variants based on different criteria for variant annotation and variant frequency. For instance, for annotation, we assessed not only protein-truncating variants, but also missense variants predicted as deleterious by various contemporary tools (e.g. AlphaMissense, REVEL, popEVE, and PrimateAI-3D)^12–15^, analyzing various combinations of the prior classes. For frequency, we applied filters restricting to ultra-rare (maximum population minor allele frequency [MAF_max_]<0.001%), rare (MAF_max_<0.1%), or rare+low-frequency variants (MAF_max_<1%). To then produce a single test statistic for a given gene, we combined the *P*-values from all masks into a single *P*-value using the Cauchy distribution test^16,17^.

### Fifteen genes with rare coding variation linked to AF risk

We observed that the mask-based test statistics from our burden analysis were well-calibrated in each of the 8 contributing datasets (**Supplementary Figure 1**) and in the meta-analysis (inflation factor at the 90th percentile of test statistic distribution [λ_90%_]=1.08, across 939,340 analyzed masks; **Supplementary Figure 2**). The subsequent gene-based Cauchy test statistics also showed reasonable calibration (λ_90%_=1.12, **Supplementary Figures 3**), and produced results across 19,841 gene-AF tests (**Supplementary Figure 2**).

**Figure 2:**
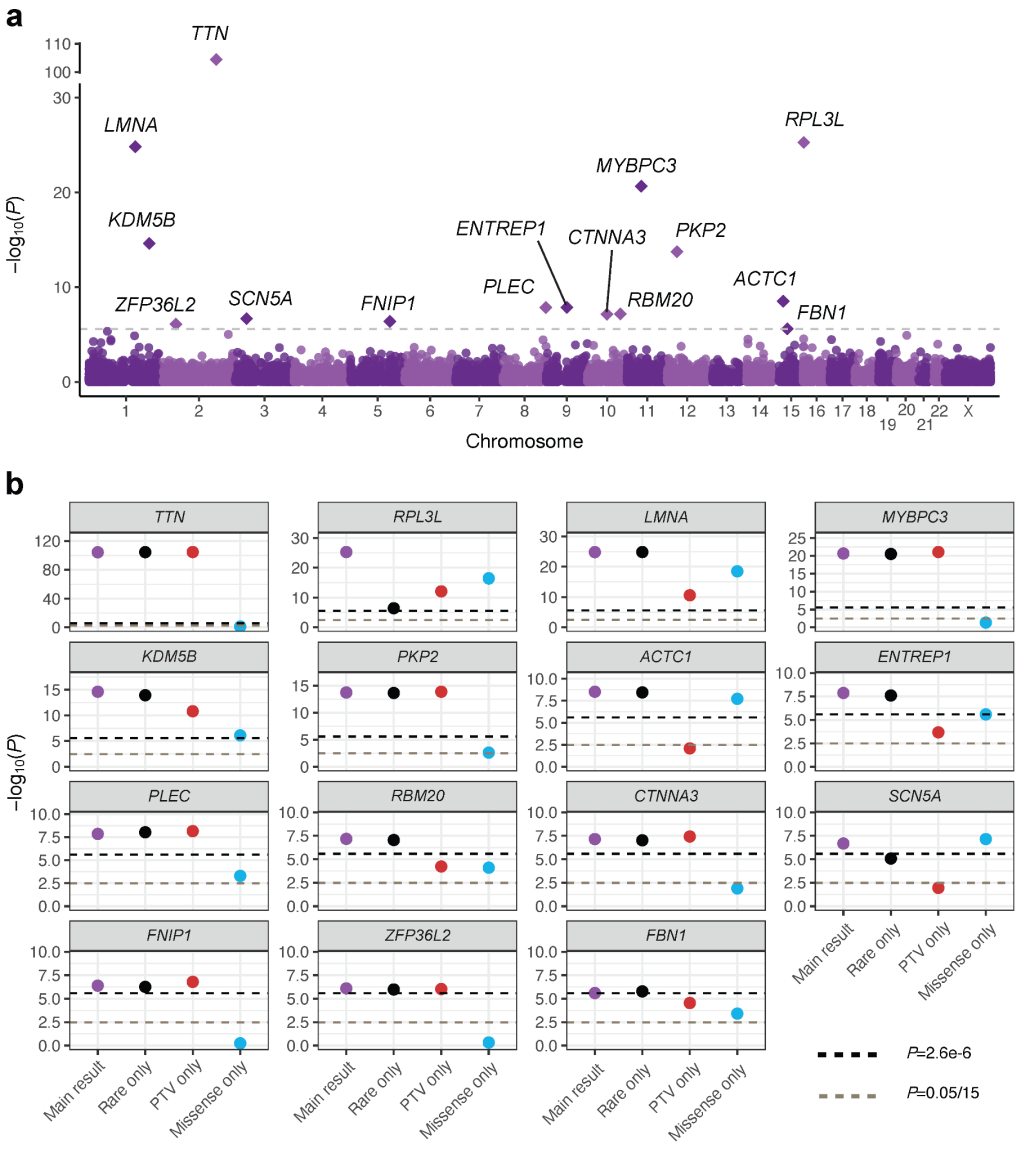
Sequencing identifies 15 genes with exome-wide significant rare variant association with AF. Part **a** is a Manhattan plot that summarizes the association results from the burden testing discovery analysis. Each dot/diamond represents a single gene, while the y-axis represents the −log10 of the Cauchy *P*-value for the given gene and the x-axis represents the genomic position of the gene. A diamond indicates that the gene reached Bonferroni-corrected exome-wide significance in the analysis; these genes are additionally annotated with the gene name. The dotted line shows the Bonferroni-corrected exome-wide significance threshold. Part **b** represents a multi-panel dot plot, showing several important sensitivity analyses performed for each of the 15 significant genes. The y-axis again represents the −log10 of the gene-based *P*-values, while on the x-axis four different analyses are shown with respective Cauchy *P*-values - the original main analysis, an analysis restricting to rare genetic variants (that is, including only masks with MAF_max_<0.1% or MAF_max_<0.001%), an analysis restricting only to PTV variant masks, and an analysis restricting only to missense variant masks. The black dotted line represents the original exome-wide significance cutoff, while the gray dotted line represents the suggestive cutoff taking into account Bonferroni correction for 15 genes assessed in the sensitivity analyses (*P*=0.05/15). Abbreviations: PTV, protein-truncating variants.

At the Bonferroni-corrected exome-wide level (α=0.05/19,841=2.5×10^−6^), we identified 15 genes significantly associated with AF (Figure 2a and Table 1). Among these, *TTN*, *RPL3L*, *LMNA*, *MYBPC3*, *KDM5B*, *CTNNA3, ENTREP1* (formerly *FAM189A2*), and *PKP2*, have been identified at exome-wide significance in previous gene-based RVAS^10^. While *SCN5A* and *PLEC* have been described for AF through single variant testing of low-frequency protein-coding variation^9,18^, no association has previously been reported through gene-based RVAS for these genes. Notably, *RBM20*, *ACTC1*, *FBN1*, *FNIP1* and *ZFP36L2* have not been described at exome-wide significance in any prior rare variant studies for AF. These results show how the expanded sample size and improved analytical approach in our study has led to several novel RVAS genes for AF.

When assessing the annotation masks contributing to the significant signals, we found that many genes were driven strongly by protein-truncating variation, notably for *TTN* and *MYBPC3* (Figure 2b and Table 1). Nevertheless, for 4 genes (*RPL3L*, *ENTREP1*, *LMNA* and *SCN5A*) missense variation yielded stronger significance than truncating variants, and for another 5 genes (*KDM5B*, *PKP2*, *PLEC*, *RBM20* and *FBN1*) we observed significant or suggestive associations for missense variants alongside the stronger signal for truncating variants (Figure 2b). No specific missense tool consistently produced the best association signal across genes (Table 1; **Supplementary Table 3**). Our results therefore indicate that rare missense variants play an important role across several AF genes, and further support a broad masking/annotation approach to burden testing^7^, as applied here.

Previous work has shown that RVAS with lenient frequency thresholds (i.e., MAF<1%) may capture LD with common genetic variants^19^. We therefore assessed the frequency thresholds used in our analysis, and found that 13 of the associations remained effectively unchanged when restricting only to truly rare variants (MAF_max_<0.1%; Figure 2b and Table 1). The association for *RPL3L* was diminished, but remained exome-wide significant (*P*=3.6×10^−7^), due to the exclusion of the low-frequency Ala75Val missense variant and c.1167+1G>A truncating variant^9,20^. The association for *SCN5A* was diminished due to the exclusion of the low-frequency p.Thr220Ile missense variant^9,21^, but still showed significance in this sensitivity analysis (*P*=8.3×10^−6^). As such, all 15 genes showed strong evidence of association with AF through truly rare coding variation.

To further scrutinize the identified associations, we performed several additional subgroup analyses (**Supplementary Table 3**). First, we performed a sensitivity analysis restricting only to individuals with genetically-inferred European ancestry (N=106,239 cases), to assess whether the identified associations were potentially driven by ancestry bias. Reassuringly, European-only test statistics showed a similar distribution to the primary analysis (**Supplementary Figure 3**), and all 15 of the identified signals showed evidence of association in the European-only analysis (all *P*<5×10^−4^). Furthermore, in sex-stratified analyses (**Supplementary Figures 4–5**), all genes showed at least nominal significance in both genetically-inferred males and genetically-inferred females. Overall, these results support the validity of the RVAS findings.

### Replication of RVAS genes

We then attempted replication of the identified signals using data from the All of Us Research Program^22^ (analyzing samples that were not included in our discovery effort; N=6,513 AF cases and N=96,848 referents; **Supplementary Note**) and using samples from the Genomics England 100,000 Genomes Program^23,24^ (N=3,387 cases and N=72,716 referents; **Supplementary Note**). Focusing on the masks that attained the lowest *P*-value in discovery, 14 of the 15 genes reached sufficient carrier counts in meta-analysis (≥20) and could be tested for association with AF (*ZFP36L2* could not be tested). Of the 14 testable genes, 7 genes significantly replicated at one-sided *P*<0.0035 (=0.05/14), including novel RVAS findings *FNIP1* (OR 8.4, *P*=4.4×10^−5^) and *PLEC* (OR 3.2, *P*=2.5×10^−3^) (**Supplementary Table 4**). A total of 10 genes showed suggestive evidence of replication (one-sided *P*<0.05), including novel RVAS gene *ACTC1* (OR 3.0, *P*=4.8×10^−3^), while 13 of 14 genes showed directional concordance with discovery (only *RBM20* was discordant; **Supplementary Figure 6**).

### Known function and clinical relevance of novel RVAS genes

Consistent with previous studies^10^, existing literature implicates the novel RVAS genes in cardiac contraction, cellular structural integrity, and/or cardiomyopathy phenotypes. *PLEC* encodes plectin, a cytoskeletal linker that maintains the structure and function of intermediate filaments in myocytes^25,26^; while *PLEC* has been proposed as a causal gene in arrhythmogenic right ventricular cardiomyopathy, strong prior evidence has been lacking^27^. *FBN1* encodes fibrillin-1, a microfibril protein that is essential for the structural integrity and elasticity of various tissues^28^; rare variants in *FBN1* are associated with Marfan’s syndrome, in which primary cardiomyopathy has been described^29^. *RBM20* (encoding a splicing-regulator of contractile genes, including *TTN*)^30^ and *ACTC1* (encoding a cardiac-specific thin filament protein) represent established monogenic cardiomyopathy genes, as they are definitively linked to dilated^31^ and hypertrophic cardiomyopathy^32^, respectively. *SCN5A* encodes the key subunit of the cardiac sodium channel, and represents the only ion channel gene identified in our analysis; while *SCN5A* is linked definitively to dilated cardiomyopathy^31^, it is also linked to many arrhythmias and represents the target of several existing AF therapies^33,34^.

While the molecular functions of *FNIP1* and *ZFP36L2* are less well described, both may be involved in mTOR signalling^35–37^, a pathway recently implicated in AF by common variant data^38^. Notably, *FNIP1* has recently been linked to a recessive syndrome of B-cell deficiency and cardiomyopathy in multiple families^38,39^, with many affected patients developing hypertrophic cardiomyopathy with pre-excitation^37^. *ZFP36L2* has recently been linked to peripartum cardiomyopathy in a murine model, with *Zfp36l2* knock-out mice developing DCM-like phenotypes during pregnancy^36^. Further studies will be needed to understand the therapeutic potential of these genes in the context of AF, and other cardiovascular disorders.

### Effect sizes conferred by rare coding variants

**Figure 3:**
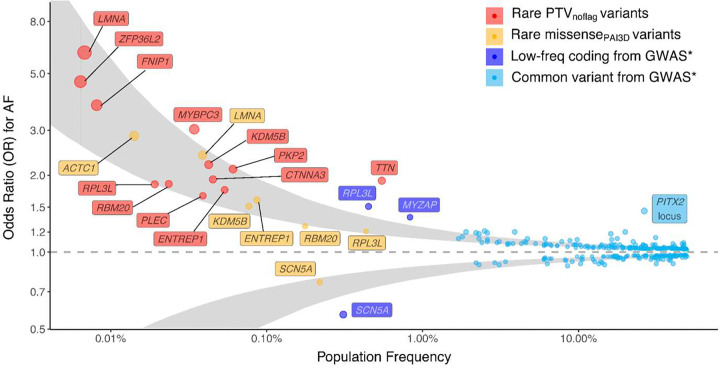
Genomic effect sizes for AF across the allele frequency spectrum. The dot plot shows the relationship between variant frequency and effect size for AF, for single variants and rare variant burdens across the allele frequency spectrum. The x-axis represents the (cumulative) minor allele frequency in the meta-analysis, while the y-axis represents the OR for AF. Red dots show PTVnoflag masks, for genes that reached significance in the RVAS discovery analysis, and for which the PTVnoflag mask reached *P*<0.05/15; gene names are highlighted in red boxes. Yellow dots indicate PrimateAI-3D predicted-damaging missense masks (missense_PAI3D_), for genes that reached significance in the RVAS discovery analysis, and for which the missense_PAI3D_ mask reached *P*<0.05/15; gene names are highlighted in yellow boxes. In dark blue, protein-coding low-frequency variants are plotted, using data from a previous AF GWAS^9^; gene names are highlighted in dark blue. In light blue, common sentinel variants from a previous AF GWAS are plotted, highlighting the well-described *PITX2* locus in light blue^9^. The gray area indicates the fitted relationship between minor allele frequency and effect size, fit through a linear regression of log(absolute observed beta) on the log(observed minor allele frequency); the extreme borders of the gray area (furthest away from zero) and the least extreme borders (closest to zero) represent the fit for the upper-bound and lower-bound of the 95% confidence interval of the observed absolute beta coefficients, respectively. Abbreviations: PTV_noflag_, protein-truncating variants without any flags; missense_PAI3D_, missense variants predicted as damaging by the primate-AI-3D algorithm; GWAS, genome-wide association study.

For 14 of the significant genes, the strongest (that is, most significant) mask was associated with a positive effect size (β>0; odds ratio [OR]>1), indicating an overall increased risk of AF conferred by rare variants for those genes. These masks showed ORs ranging from 1.2 to 4.4, with 13 genes showing an OR larger than 1.6 (Table 1). These effects are perhaps smaller than would be expected by a highly-penetrant pathogenic variation, supporting a “multiple hit” concept for AF development in people with an inherited predisposition. In comparison, using data from a recent AF GWAS^9^, we found that most AF-associated common variant loci showed effect sizes with OR<1.3, with the well-known *PITX2* locus showing an OR of 1.6 (Figure 3 and **Supplementary Table 5**). These findings highlight the spectrum of effects conferred by genetic variants across the frequency spectrum.

Interestingly, the strongest mask for *SCN5A* showed a negative effect estimate (OR 0.8, 95%CI [0.7; 0.9]), indicating that the qualifying variants generally conferred protection against AF. Notably, the protective effect seemed driven by rare missense variants (partly but not exclusively due to the p.Thr220Ile variant^9,21^), as protein-truncating variants were nominally associated with increased risk of AF (OR_LOF_ 1.8, 95%CI [1.2; 2.8]; *P*_Cauchy_=0.01). We speculate that different variant classes confer different functional consequences, or that sodium channel dysfunction may have both antiarrhythmic and proarrhythmic effects on atrial arrhythmia^40^, potentially dependent on the degree of inhibition/channel loss. More broadly, these findings highlight the utility of our extensive masking procedure to identify diverging effects across variant classes.

Indeed, our dataset allowed us to estimate and compare effects across different annotation classes and genes. We found that truncating variants in several of the associated genes conferred substantial effect sizes on AF. For instance, high-confidence truncating variants in *LMNA*, *ZFP36L2*, *FNIP1* and *MYBPC3* all conferred over 3-fold increased odds of AF, while truncations of *KDM5B* and *PKP2* were associated with over 2-fold odds (Figure 3). Focusing on variants predicted to be deleterious by PrimateAI-3D^9,15^, we found that missense variant masks in certain genes were associated with substantial effect sizes (OR>2), in particular for *LMNA* and *ACTC1* (Figure 3). Of note, missense variants, even those predicted as damaging, will have widely varying functional effects and some might still be benign^41,42^, suggesting that some subset of missense variants in these genes may in fact confer even larger effects on AF.

We then combined the effect sizes estimated in our analysis with data from a previously-published GWAS focused on common and low-frequency variants^9^ (**Supplementary Table 5**). Modeling the absolute effect sizes against the observed allele frequencies, we found a strong relationship where rarer variants are associated with larger effect sizes (Figure 3). Taken together, our effect size data are consistent with negative selection driving high-effect alleles to low population frequency, and highlight the utility of RVAS to identify AF genes with large biological magnitude.

### Convergence of rare and common variant studies for AF

**Figure 4:**
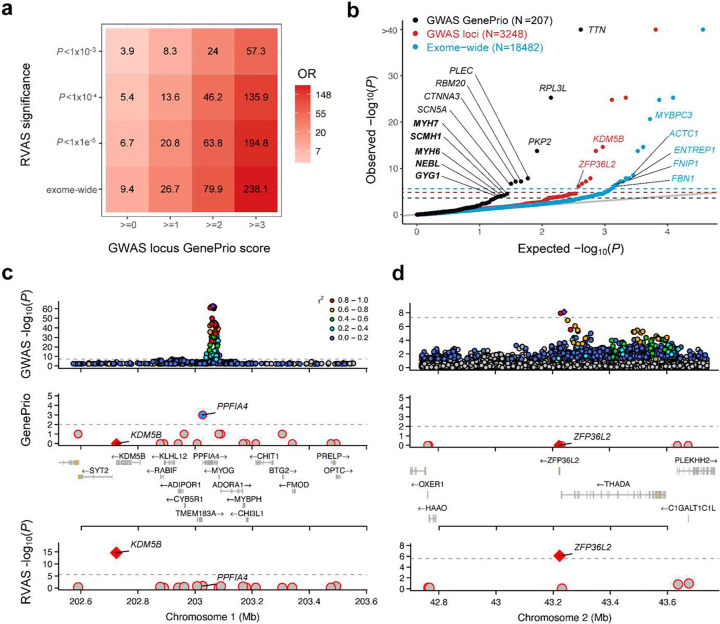
Integrated gene prioritization for AF from rare and common variant studies. Panel **a** is a heatmap showing Fisher exact test enrichments of RVAS genes and GWAS genes. The y-axis shows different significance thresholds (based on gene-based Cauchy *P*-values) to determine RVAS genes from the discovery analysis, while the x-axis shows different cutoffs for GenePrio scores for genes near loci from a recent AF GWAS^9^ (where GenePrio score of 0 includes any gene within 500kb from genome-wide significant loci). The cell values show ORs from Fisher exact tests. Panel **b** is a quantile-quantile plot showing results from our RVAS discovery analysis, with expected −log10 *P*-values on the x-axis and observed gene-based −log10 Cauchy *P*-values on the y-axis. The black dots represent Cauchy *P*-values for genes residing within GWAS loci that attained GenePrio scores >=2; red dots represent Cauchy *P*-values for all genes residing within GWAS loci; blue dots represent Cauchy *P*-values for all assessed genes exome-wide. Among the black dots, all genes surpassing the Bonferroni-corrected significance threshold (*P*<0.05/207; shown in black dotted line) are annotated with their gene name, with genes not identified in the exome-wide discovery analysis highlighted in bold; among the red dots, genes surpassing the Bonferroni-corrected threshold (*P*<0.05/3248; red dotted line) are annotated with the gene name if they were not among the genes with high GenePrio score; among the blue dots, genes surpassing the exome-wide Bonferroni-corrected threshold (*P*<0.05/18482; black dotted line) are annotated with gene names if the genes were not near any GWAS loci. Of note, analyses in panels **a** and **b** are restricted to autosomal genes. Panel **c** shows a LocusZoom^64^ plot for the *KDM5B*/*PPFIA4* locus from AF GWAS^9^; the top track shows single variants with −log10 *P*-values on the y-axis and genomic position on the x-axis (with colour indicating LD with respect to the sentinel variant in the locus); the middle track shows genes in the locus with GenePrio scores on the y-axis and genomic position on the x-axis; and the bottom track shows genes in the locus with RVAS Cauchy *P*-values (from the discovery analysis) on the y-axis and position on the x-axis. Panel **d** is a similar LocusZoom plot showing data for the *ZFP36L2* locus. Abbreviations: RVAS, rare variant association study; GWAS, genome-wide association study; Mb, megabases.

In previous work, we showed a weak convergence of RVAS and GWAS genes for AF, although this analysis was based on a relatively underpowered RVAS^10^. We therefore reassessed this question, by utilizing our improved RVAS data and common variant data from a recent AF GWAS^9^. We found that genes residing near significant GWAS loci were approximately 9.4-fold enriched for exome-wide significant genes from our RVAS (*P*=3.5×10^−5^; 95%CI [2.93; 35.1]; Figure 4a and **Supplementary Tables 6–7**). Most of the genes found near GWAS loci may, however, not represent causal genes^4,43^, and therefore we also assessed genes prioritized from AF GWAS using the GenePrio algorithm^9^. With increasing GenePrio prioritization stringency, we observed increasing RVAS gene enrichment (Figure 4a), where genes with GenePrio score >=3 reached an over 200-fold enrichment for exome-wide RVAS genes (OR 231, 95%CI [67.43; 796.85]; *P*=3.2×10^−12^; **Supplementary Table 7**).

To evaluate whether the observed enrichment was driven by LD between GWAS and RVAS variants, we performed a sensitivity analysis in which we restricted to RVAS data based on rare variants only (MAF_max_<0.1%); in this sensitivity analysis, we observed highly similar patterns albeit with somewhat depleted ORs (**Supplementary Figure 7a** and **Supplementary Table 7**). Building on our previous findings^10^, we now demonstrate a marked convergence between GWAS and RVAS genes for AF, especially for GWAS genes with high bio-informatic prioritization.

### Integrated gene prioritization for AF from common and rare variation

Despite strong convergence, not all RVAS genes were explicitly prioritized by the existing GWAS data. Of the 15 significant RVAS genes, 9 resided near GWAS loci, of which 7 reached high GenePrio scores (Figure 4b and **Supplementary Figure 7b**). Notably, *KDM5B* and *ZFP36L2* both resided near GWAS loci but attained GenePrio scores of 0. In the *KDM5B* locus (Figure 4c), the gene nominated by GenePrio was *PPFIA4*, encoding a protein expressed primarily in brain^44,45^. In the *ZFP36L2* locus, no gene attained high prioritization through GenePrio (Figure 4d). These results indicate that RVAS data may aid in accurate gene prioritization from GWAS loci, in certain cases.

Conversely, given the relatively limited detection power of rare variant testing, we then used GWAS data to narrow the analytical search space for RVAS. Specifically, we assessed whether additional significant signals could be identified from our RVAS data by subsetting only to genes residing within GWAS loci (N=3248 genes; alpha=1.5×10^−5^) or GenePrio-prioritized genes (N=207 genes; alpha=2.4×10^−4^). Among the 207 GenePrio genes, we identified 5 significant RVAS genes that were not identified previously at exome-wide significance (*MYH7*, *SCMH1*, *MYH6*, *NEBL* and *GYG1*; Figure 4b and **Supplementary Figure 8**). Of these, we found that *MYH7* and *MYH6* remained significant when restricting only to rare variation (MAF_max_<0.1%; **Supplementary Figure 7c** and **Supplementary Table 3**). Both genes reside within the same GWAS locus, in which *MYH6* was nominated by GenePrio as the most likely causal gene (**Supplementary Figure 8a**). In contrast, our RVAS data indicate that both *MYH6* and *MYH7* may play a causal role in AF pathogenesis, consistent with the expression patterns of the proteins encoded by these genes (atrial- and ventricular-specific myosin heavy chain proteins, respectively)^46^.

Associations for *SCMH1*, *NEBL* and *GYG1* became insignificant after restricting the RVAS to rare variants only (**Supplementary Figure 7c** and **Supplementary Table 3**). These genes may have been strongly driven by bona fide effects from low-frequency coding variation, as is likely for *NEBL* (through the low-frequency Tyr89Ter truncating variant; **Supplementary Figure 7c**). Alternatively, they may have been affected by LD between GWAS variants and low-frequency variants included in the RVAS^19^ (plausible for *SCMH1* and *GYG1*). These findings further underscore the importance of frequency-based sensitivity analyses in RVAS.

### Contribution of rare protein-disrupting variation to AF heritability

It remains unclear to what extent rare genetic variation, including rare coding variation, contribute to AF risk in the population. To this end, we aimed to estimate the heritability attributable to rare protein-disrupting variation using Burden Score Heritability Regression (BHR)^8^. We initially applied BHR to unrelated European-ancestry individuals from the UK Biobank (N=36,006 AF cases and 385,851 referents), as the UK Biobank represents the largest population-based dataset with WGS across a relatively homogeneous ancestry group^47^.

**Figure 5:**
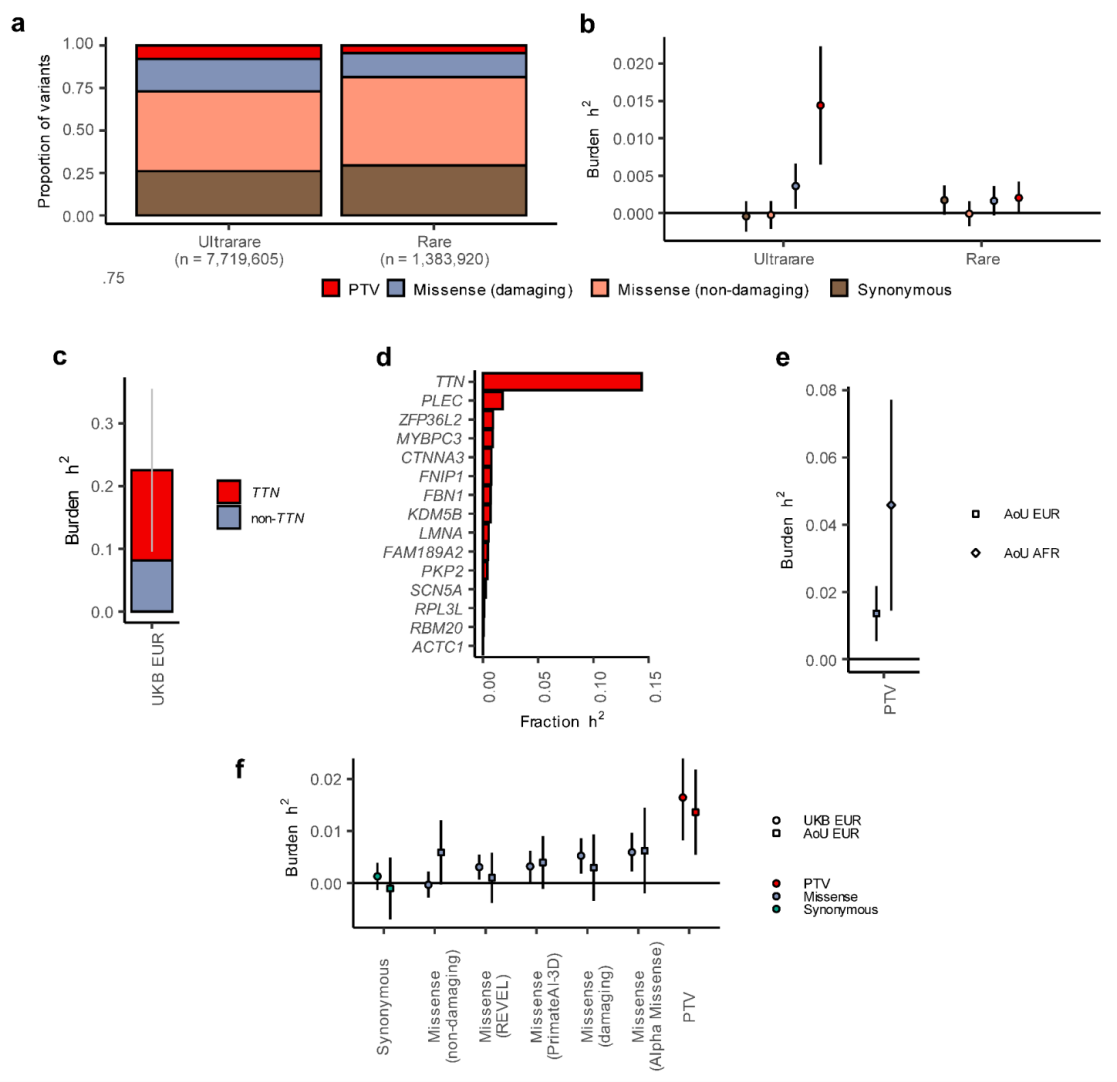
Burden score heritability regression unveils the contribution of rare protein-disrupting variation to AF susceptibility. Panel **a** is a stacked bar chart showing the proportions of different annotation classes among the variants included in Burden Score Heritability Regression (BHR) in the UK Biobank analyses, with the left bar showing ultra-rare variants and the right bar showing rare variants. Red indicates PTVs, blue indicates predicted-damaging missense variants, peach indicates predicted non-damaging missense variants and brown indicates synonymous variants. Panel **b** is a dot plot showing results from the BHR analysis in the UK Biobank, with estimated liability-scale h^2^_burden_ for AF on the y-axis, and different masks (split by annotation and frequency) on the x-axis. Error bars represent 95% confidence intervals. The colour scheme is identical to panel a. Panel **c** is a stacked bar chart showing proportions of h^2^_burden,PTV_ for AF in UK Biobank, as explained by *TTN* and the remaining 14 significant genes from our gene-based discovery analyses. Red indicates the proportion explained by *TTN*, and blue indicates the proportion explained by the remaining 14 genes. The error bars represent the 95% confidence interval for the proportion explained by all 15 genes. Panel **d** is a bar chart showing the proportion of h^2^_burden,PTV_ for AF in UK Biobank, as explained by each of the 15 significant genes. Each bar on the y-axis is a different gene, while the x-axis shows the proportion of h^2^_burden,PTV_. Panel **e** is a dot plot showing h^2^_burden,PTV_ for AF from analysis in the All of Us Research Program, as estimated within European ancestry individuals (left) and African ancestry individuals (right). The error bars represent 95% confidence intervals. Panel **f** is a dot plot with h^2^_burden_ estimates for AF from both the UK Biobank and the All of Us Research Program, with different annotation classes on the x-axis. Color schemes match the color schemes from panels a and b. Abbreviations: h2, heritability; PTV, protein-truncating variants; UKB, UK Biobank; AoU, All of Us Research Program; EUR, European genetic ancestry; AFR, African genetic ancestry.

Across the protein-coding regions of the genome, approximately a quarter of the variants represented synonymous variants conferring no change to the protein sequence^48^; approximately half of the variants represented missense variants predicted to be benign by 4 prediction tools^12–15^; 10–15% represented missense variants predicted to be damaging by at least one tool; and a small proportion (<5%) represented high-confidence protein-truncating variants (Figure 5a and **Supplementary Table 8**).

We then performed BHR in the UK Biobank dataset, stratifying variants by annotation and frequency bin (Methods). Ultra-rare (MAF<0.001%) truncating variants were significantly enriched for AF heritability, explaining approximately 1.4% of AF susceptibility on the liability-scale (*P*=3.6×10^−4^; 95%CI [0.65%; 2.2%]; Figure 5b and **Supplementary Table 9**). Together, ultra-rare and rare (MAF<0.01%) truncating variants explained 1.6% of variation in AF risk (*P*=9.4×10^−5^; 95%CI [0.82%; 2.5%]; **Supplementary Table 10**). Ultra-rare missense variants, predicted as damaging, also conferred significant burden heritability (h^2^_burden_ = 0.36%, 95%CI [0.057%; 0.66%]; *P*=0.020; Figure 5b), with ultra-rare and rare damaging missense variants together explaining 0.53% of variance in AF susceptibility (95%CI [0.18%; 0.87%]; *P*=2.5×10^−3^; **Supplementary Table 10**). In contrast, synonymous variants and benign missense variants were not significantly associated with AF heritability (Figure 5b).

We note that BHR assumes that variant effects are homogeneous within gene masks^8^. Therefore, given limitations of missense prediction tools and given variability in phenotypic effects conferred by damaging missense variants^41,42^, our missense variant estimates likely represent lower-bounds of the true additive heritability. As such, the BHR results indicate that rare protein-disrupting variants explain at least 2% of AF susceptibility in the middle aged population, with additional heritability from missense variants being probable.

We then assessed what proportion of rare coding variant heritability could be explained by known AF genes. Focusing on protein-truncating variants, we found that approximately 23% of the estimated h^2^_burden_ could be attributed to variants affected the 15 genes identified in our RVAS (Figure 5c and **Supplementary Table 11**). When assessed by gene, *TTN* clearly explained the largest proportion of h^2^_burden_ (14%; Figure 5d), consistent with the important role of *TTN* in AF and the relatively high population frequency of *TTN* truncations^49^. While these findings demonstrate that substantial rare variant heritability for AF is concentrated in a small number of genes, as previously shown for other traits^8^, they also indicate that over 65–80% of h^2^_burden_ may lie in rare variants affecting yet unknown AF genes.

While BHR has been applied successfully to complex traits in UKB before, the replicability of masks across cohorts and ancestries remains unexplored. To replicate our BHR findings, therefore, we then analyzed samples from the All of Us Research Program^22^ (Figure 5ef and **Supplementary Figure 9**). Assessing individuals of European genetic ancestry (N=14,816 AF cases and N=148,109 referents), we recapitulated highly similar h^2^_burden_ estimates across various annotation classes (Figure 5f and **Supplementary Table 10**). Notably, rare and ultra-rare truncating variants explained 1.4% of AF susceptibility on the liability-scale (95%CI [0.054%; 2.2%]; *P*=1.1×10^−3^), while synonymous variants showed no signal (Figure 5f).

Finally, we assessed other ancestry groups within the All of Us dataset. Requiring at least 2,000 unrelated AF cases, we attained sufficient sample size to perform BHR among individuals of genetically-inferred African ancestry (N=2,576 AF cases and N=51,211 referents; **Supplementary Figure 9b**). Among African-ancestry individuals, we also observed a significant enrichment of AF heritability from rare and ultra-rare truncating variants (h^2^_burden_ = 4.6%, 95%CI [1.5%; 7.7%]; *P*=4.1×10^−3^), without significant signal for synonymous or missense variant classes (Figure 5e and **Supplementary Table 10**). Despite a numerically larger point estimate for h^2^_burden_ as compared to European ancestry, we note that confidence intervals were large and overlapping with the European-ancestry estimates. Taken together, our findings indicate that rare protein-disrupting variants contribute significantly to AF heritability across European and African ancestry, explaining at least a few percent of population variance.

## Discussion

In this study, we leveraged one of the largest sequencing case-referent datasets for any disease to date, consisting of over 123,000 AF cases and over 861,000 referents, with replication in an additional 10,000 cases and 170,000 referents. Through RVAS we identified 15 significant genes, including several not previously linked to the arrhythmia. While rare protein-disrupting variants conferred effect sizes far exceeding typical effects of common genetic variation, we showed strong convergence between rare and common variant associations, indicating shared biological mechanisms across the allele frequency spectrum. At the same time, rare variant analyses refined causal inference at certain GWAS loci and highlighted novel genes overlooked by previous GWAS. We subsequently estimated the contribution of rare coding variants to disease heritability, demonstrating that rare protein-disrupting variants explain a measurable fraction (at least 2%) of AF susceptibility across European and African ancestries. Importantly, the 15 exome-wide significant genes explain only a minority of this heritability, indicating that additional RVAS genes may be discovered in future endeavors of even larger size. Together, these findings provide a comprehensive view of AF genetic architecture. More broadly - while highlighting the massive sample sizes required for RVAS of common diseases - our results illustrate the complementarity of RVAS and GWAS for disease gene discovery and establish a framework for linking rare coding variation to complex disease biology at scale.

## Methods

Rare variant association meta-analysis

### Study participants

To study the rare coding variant architecture of AF, we combined data from various sets where both AF status and sequencing - either whole-exome (WES) or whole-genome (WGS) - were available. The included cohorts used various study designs and/or ascertainment procedures, including i) case-control efforts aimed specifically on the genetics of AF or early-onset AF, ii) population-based / longitudinal cohorts, iii) health system-based cohorts, and iv) clinical trials within AF or other patient populations.

Specifically, in our effort, we included:

- WGS data from the full UK Biobank cohort^47^ (N=40486 AF cases and N=447431 referents)
- WES data from a large case-control assembly from the Centers of Common Disease Genomics^10^ (CCDG; N=34586 AF cases and N=52714 referents; 19 cohorts including MGB, ENGAGE AF-TIMI 48, PEGASUS-TIMI 54, SAVOR-TIMI 53, BioVU-AF, MGH-AF, TMDU-AF, TCAI-AF, SWISS-AF, DECAF, UWO, VAFAR, AFNET-EAST, UCSF-AF, GENAF, GGAF, RACE 3/5, UIC-AF, and BioHEART)
- WES data from the Geisinger Health System MyCode study^50^ (N=19733 AF cases and N=133605 referents)
- WGS data from a large case-control assembly from the Trans-Omics for Precision Medicine Program and CCDG^10^ (TOPMed-CCDG; N=11166 AF cases and N=25806 referents; 28 cohorts including ARIC, BioMe, BioVU, CCAF, CFS, CHS, COPDGene, AustralianFamilialAF, CATHGEN, FHS, GGAF, HVH, JHS, MGH-AF, MMP-AF, AFLMU, Partners, PMBB-AF, DECAF, UCSF-AF, miRhythm, VU-AF, VAFAR, WGHS, GENAF, JHU-AF, INSPIRE-AF)
- WGS data from freeze 7 of the All of Us Research Program^5,51^ (N=10264 AF cases and N=158096 referents)
- WES data from additional samples from MGB (Massachusetts General Brigham Biobank)^5,52^ that were not sequenced as part of CCDG-WES (N=4369 AF cases and N=17564 referents)
- WES data from the FOURIER (TIMI 59) clinical trial^53^ (N=1394 AF cases and N=14597 referents)
- WES data from the DECLARE-TIMI 58 clinical trial^54^ (N=1108 AF cases and N=11302 referents).

Baseline characteristics for the 8 discovery datasets are presented in **Supplementary Table 1**, while detailed cohort descriptions are presented in the **Supplementary Note**. Replication analyses were performed using WGS data from freeze 8 of the All of Us Research Program, as well as data from the Genomics England 100,000 Genomes Project (**Supplementary Note**).

All participants provided written informed consent, and participating studies obtained ethical approval from institutional review boards where appropriate. The UK Biobank resource was approved by the UK Biobank Research Ethics Committee and all participants provided written informed consent to participate. Use of UK Biobank data was performed under application numbers 17488 and 176602, and the study protocol was approved by the Massachusetts General Brigham Institutional Review Board. Use of All of Us data was approved through agreements of Massachusetts General Brigham and Amsterdam UMC with the All of Us Research Program.

### Sequencing and quality control

Details on sequencing are provided in the **Supplementary Note**. In each dataset, appropriate quality control was applied, which in most cases included extensive genotype-level, variant-level and sample-level quality control on top of any central filters that may have been applied. Details of the quality control steps are also provided in **Supplementary Note**. Notably, to avoid inflation in our meta-analysis arising from duplicated samples across datasets, we took several steps: Where possible, we combined the individual-level data to identify potentially overlapping samples (possible for TOPMed-CCDG, CCDG-WES, MGB, FOURIER, and DECLARE; **Supplementary Note**). We further removed samples from the All of Us datasets that had ZIP codes from Massachusetts, given known sample overlap between All of Us Research Program and MGB^5^.

### Variant annotation

In all datasets, we annotated genetic variants using the Variant Effect Predictor (VEP)^55^ with the LOFTEE plugin^56^ (https://github.com/konradjk/loftee), as well as the dbNSFP database (https://www.dbnsfp.org/)^57^ and various additional prediction tools (**Supplementary Note**). Our annotation pipeline first used VEP to identify rare coding variants affecting the ENSEMBL canonical transcripts of protein-coding genes, and then extracted missense variants (annotated as such by VEP) and high-confidence protein-truncating variants (PTVs; annotated as such by LOFTEE). Among PTVs, we distinguished two groups, including one irrespective of potential LOFTEE flags, and a more stringent group excluding PTVs with any LOFTEE flags^56^ (PTVnoflag; **Supplementary Note**). Among missense variants, various bioinformatic prediction tools were utilized to identify groups of variants with potentially damaging effects, namely PrimateAI-3D, REVEL, popEVE, and AlphaMissense (**Supplementary Note**)^12–15^.

We also annotated genetic variants using ancestry-specific frequencies from gnomAD v2, focusing on frequencies within non-Finnish European, African, South-Asian, East-Asian and Admixed-American super-populations^56^. In most datasets, we then annotated each genetic variant with the maximum population minor allele frequency (MAF_max_) representing the highest allele frequency across any of the gnomAD super-populations and the in-sample frequency in the given dataset. For the Geisinger MyCode dataset, the MAF_max_ was computed simply as the in-sample frequency.

### Masking strategy for rare variant testing

In gene-based burden testing, genetic variants are generally pooled to improve statistical power for rare variation. However, the optimal strategy for pooling of variants, or masking, is not known and likely not uniform across protein-coding genes^7,19^. From the various PTV, missense and frequency annotations, we therefore created many masking combinations, as shown in Figure 1.

Based on PTVs only, we created up to two masks for each gene (PTV and PTVnoflag). For missense variants, for each prediction tool, we created a mask that included missense variants predicted as damaging by the given tool; we also created a mask that included missense variants predicted as damaging by any one of the four tools (miss1/4), and a strict masks including missense variants predicted as damaging by at least three of the four tools (miss3/4); as such, up to 6 missense masks were created per gene. We also created masks combining both PTV masks with each of the missense masks, yielding 2×6=12 additional PTV+missense masks. In total, therefore, 20 different masking schemes were created based on variant annotation.

In our masking, we also applied various filters determined by variant frequency. We essentially created masks for ultra-rare variants (MAF_max_<1×10^−5^), masks for rare variants (MAF_max_<1×10^−3^), and masks for rare and low-frequency variants (MAF_max_<0.01). As such, for any given protein-coding gene, up to 20×3=60 different masks were created based on annotation and frequency.

### Burden association analyses

To identify associations between AF status and rare variant masks, we performed rare variant burden analyses within each dataset, using various versions of REGENIE^58^. In all REGENIE analyses, we first ran step 1 using common genetic variation to fit logistic null-models, and applying the leave-one-out-cross-validation approach to construct the REGENIE stacked regressors/PRSs. In step 2, we then performed the burden association tests, using logistic regression models applying approximate-Firth’s correction - with back-correction of SEs - to account for data imbalances. In both step 1 and step 2, at minimum, covariates were included to account for age, sex, and principal components of ancestry (and the REGENIE PRS in step 2)^58,59^; additionally, dataset-specific covariates were applied in some cases. After step 2, burden results were filtered based on the cumulative minor allele count (cMAC) of the masks, to avoid spurious results driven by low counts; for datasets with relatively well-balanced case-control ratios we required cMAC>=5 (CCDG-WES, TOPMed-CCDG), while for other datasets we required cMAC>=10; in the All of Us dataset we required carrier count>=20 to comply with biobank policies. Details on data sources, common variant filtering, covariates, and mask filtering are provided in **Supplementary Table 2**.

Of note, for all autosomal genes, the above approach was applied as described, combining both genetically-inferred males and genetically-inferred females in a single analysis. However, for genes residing on chromosome X, analyses were performed in males and females separately.

To then combine the results from the various datasets, we performed a meta-analysis. An initial inverse-variance-weighted fixed-effects meta-analysis - implemented in METAL^60^ - was first applied to compute meta-analytical beta coefficients, meta-analytical SEs, and meta-analytical *P*-values, from the dataset-specific beta coefficients and SEs. METAL was also used to compute heterogeneity Q-values and associated *P*-values. Despite accounting for data imbalance within each dataset using Firth’s corrections, it has been shown that test statistics can become slightly miscalibrated in the meta-analytical setting for rare variants^61^. We therefore applied a genotype-count based saddlepoint approximation method to produce adjusted meta-analysis *P*-values^61^, using the study-specific cMAC values and *P*-values as input; the saddlepoint approximation was applied to any mask reaching nominal *P*<0.05 within the regular fixed-effects meta-analysis. After meta-analysis, we filtered results down to masks reaching meta-analytical cMAC>=20, to avoid spurious results driven by low counts. We also filtered results down to masks where at least two datasets contributed to the meta-analysis, to avoid signals driven entirely by singular datasets.

### Cauchy combination

The above meta-analysis would then produce up to 60 different mask-AF association results for each gene (and up to 120 for X-chromosomal genes; 60 in males and 60 in females). To then produce a single test statistic for a gene, we used the Cauchy distribution test^16^ in a layered approach^17^. The Cauchy distribution allows for valid aggregation of many potentially correlated *P*-values, into a single omnibus *P*-value^16^. In our layered approach, for a given gene, we first combined *P*-values from all qualifying missense-only masks into a single ‘missense *P*-value’ using the Cauchy distribution; a similar approach was applied to combine PTV-only masks into a ‘PTV *P*-value’, and PTV+missense masks into a ‘PTV+missense’ *P*-value. Then, the missense Cauchy *P*-value, the PTV Cauchy *P*-value and the PTV+missense Cauchy *P*-value were combined in a second layer to produce the final Cauchy *P*-value for the given gene. For X-chromosomal genes, the Cauchy approach was applied separately for males and females, therefore producing two final Cauchy *P*-values (one for males and one for females). All association and Cauchy *P*-values are two-sided unless otherwise specified; multiple testing correction was applied using the Bonferroni-correction.

### Sensitivity analyses

For autosomal genes, sensitivity analyses were performed restricting to genetically-inferred males only, and to genetically-inferred females only. For all genes, sensitivity analyses were performed in which analyses were restricted only to individuals with genetically-inferred European ancestry, to assess whether any identified signals were driven by ancestry bias in our pan-ancestry analysis. Several other sensitivity analyses were performed to assess the contribution of various annotation and frequency bins to the identified signals, including analyses restricting only to rare variation (that is, including only masks with MAF_max_<0.1% or MAF_max_<0.001%), as well as analyses restricting only to missense masks or only to PTV masks.

### Integrated analyses of rare and common genetic variation

#### Effect sizes across the frequency spectrum

To study the relationship between variant frequency and effect size for AF, we combined our rare variant association data with data from a recent large-scale GWAS for AF that included over 180,000 AF cases^9^. We first extracted high-quality common variants from their data: From the curated list of 354 genome-wide significant sentinel variants with MAF>1%, we further restricted to variants where at least 2 studies contributed to the meta-analysis and to variants where at least 70% of the maximum case count contributed to the meta-analysis, leaving 327 high-quality index common variants. We also extracted data for index low-frequency variants (1%>MAF>0.1%), restricting to sentinel variants with *P*<1×10^−8^ that also represented the top variant in a given locus, and where at least two studies contributed to the meta-analysis, leaving 8 low-frequency variants; we additionally restricted to coding variants only, leaving 3 low-frequency coding variants (*MYZAP* p.Q254P, *RPL3L* c.1167+1G>T, SCN5A p.T220I). The curated common and low-frequency variants are presented in **Supplementary Table 5**.

To model variant effect sizes, we then combined the common and low-frequency variant data with our rare variant association data. For rare variant association data, we used genes that reached exome-wide significance in our discovery analyses, and we then extracted results for the PTVnoflag mask with MAF_max_<0.1% - if the PTVnoflag mask reached *P*<0.05/15 - given that the PTVnoflag mask would yield effect sizes for the most stringent subset of high-confidence PTVs. For missense variants, we used the mask including missense variants predicted as damaging by PrimateAI-3D with MAF_max_<0.1% (again, if the mask reached *P*<0.05/15).

We then fit models describing the relationship between (cumulative) MAF and absolute effect size across the common variants, low-frequency variants, PTVnoflag rare variant burdens, and PrimateAI-3D missense variant burdens. We used a logarithmic power-law model^62,63^, in which the logarithm of the absolute beta coefficient is linearly regressed on the logarithm of the minor allele frequency. To model upper-bounds and lower-bounds, we used the same approach but inputting the lower-bound of the 95% confidence interval of the absolute beta coefficient of each variant/burden, and the upper-bound of the 95% confidence interval, respectively.

#### Convergence of GWAS and RVAS genes

We aimed to quantify the convergence of genes nominated from common variant GWAS and gene-based RVAS. To this end, we utilized common variant data - and gene prioritization data - from a recent AF GWAS^9^. Notably, the study used a scoring algorithm based on different lines of evidence to score genes near GWAS loci (ranging from 0–5), assigning genes with score>=2 as more likely causal genes. To then estimate gene convergence, we used Fisher exact tests that estimated the enrichment of genes identified in our RVAS discovery analysis among GWAS-nominated genes. We assessed various cutoffs for RVAS significance (exome-wide, *P*<1×10^−5^, *P*<1×10^−4^, *P*<1×10^−3^) and various cutoffs for GenePrio score (>=0, >=1, >=2, >=3), with GenePrio>=0 including any gene within 500kb from genome-wide significant loci. Since our RVAS analysis included variant masks with MAF_max_<1%, which might show LD with common variants^19^, we performed sensitivity analyses restricting the RVAS to masks with rare variants (MAF_max_<0.1% and MAF_max_<0.001% masks).

#### Gene prioritization from RVAS and GWAS

We then assessed various ways in which RVAS and GWAS may supplement each other for gene nomination. First, we assessed exome-wide significant RVAS genes that were among GWAS loci but were not prioritized by the GenePrio algorithm. For these genes, we created LocusZoom plots^64^ - assessing the local single variant association structure, the genes residing within the locus, the GenePrio scores for all nearby genes, and the RVAS Cauchy *P*-values for all nearby genes - to identify discrepancies between RVAS and GenePrio prioritization. Second, we assessed whether GWAS could aid as a first tool to minimize the RVAS search space. To this end, we identified genes from our RVAS results, at more lenient levels of statistical significance, based on whether the genes were nominated by GWAS. We initially assessed any gene near genome-wide significant GWAS loci (correcting the RVAS *P*-value threshold for this number of genes) and then assessed only genes within GWAS loci attaining GenePrio scores >=2 (again, correcting the *P*-value threshold for this number of genes).

### Rare variant heritability

#### Burden score Heritability Regression

We used Burden Score Heritability Regression (BHR; version 0.1.0) to estimate the burden heritability for AF^8^. Briefly, burden heritability represents an estimator of the aggregate phenotypic variance explained by rare variants exome-wide (or genome-wide) assuming that rare variants within predefined groups (ie burdens or masks) have the same phenotypic effects. Burden heritability can be estimated by regressing mask-trait association statistics on gene burden scores, where the heritability estimate is proportional to the regression slope, while population stratification and any residual relatedness affect the intercept.

To estimate accurate population variances, we applied BHR to WGS data from UK Biobank (UKB) and to All of Us Research Program (AoU), as these represent relatively unselected cohorts with respect to AF phenotype. In UKB we analyzed EUR ancestry as a primary analysis, while EUR and AFR ancestries were analyzed in AoU as replication purposes (we required at least 2000 cases in a given ancestry group for this analysis, meaning we did not retain sufficient samples for other ancestries). One individual from pairs with KING-robust > 0.0884 were excluded from the BHR analysis, as to avoid inflation from relatedness. In AoU, saliva samples were excluded to exclude potential batch effects.

BHR was run exome-wide, by aggregating protein-coding variants into masks. Variants were categorized as ultrarare (allele frequency < 1 × 10^−5^) or rare (1 × 10^−5^ - 1 × 10^−4^), and variants with maximum population allele frequency > 1 × 10^−4^ were excluded. Variants were categorized as PTV, damaging missense, non-damaging missense, or synonymous variants. When missense variants were not deemed as damaging by any of the missense variant prediction tools[cite], these were defined as non-damaging missense variants. For any given gene, masks with cMAC < 20 were excluded.

After the above masking procedure, BHR was run separately for each functional category and allele frequency bin combination, after which statistics were aggregated. We used the fixed_genes option so that BHR treated the 15 significant genes from our discovery meta-analysis as fixed-effects variables. We used the genomewide_correction option to condition on the genome-wide burden in the model. Finally, we calculated the liability-scale burden heritabilities from the observed scale heritabilities, assuming an AF population prevalence of 5.30% for AoU EUR and 2.17% for AoU AFR^8,65^. We used the sample prevalence as the population prevalence for UKB EUR as UKB is a population-based cohort.

## Supplementary Material

Supplementary Files

This is a list of supplementary files associated with this preprint. Click to download.

• AFsequencingfreeze2supplement.pdf

• AFsequencingfreeze2SupplementaryTables.xlsx

## Figures and Tables

**Table 1.** Detailed results and sensitivity analyses for genes associated with AF

Gene	Cauchy *P*-value	Best mask			Sensitivity analysis *P*-values			
		Mask name	OR [95%CI]	cMAC	MAF_max_ <0.1% only	PTV only	Missense only	EUR ancestry only
*TTN*	3.72E-105	PTV+miss3/4 MAF_max_<1e-05	2.5 [2.3; 2.7]	5788	2.48E-105	1.86E-105	7.55E-02	1.84E-83
*RPL3L*	5.23E-26	PTV+miss1/4 MAF_max_<0.01	1.2 [1.2; 1.3]	31593	3.59E-07	7.41E-13	3.57E-17	3.83E-24
*LMNA*	1.50E-25	PTVnf+missREVEL MAF_max_<1e-05	4 [3.1; 5.1]	530	1.43E-25	2.36E-11	3.38E-19	5.17E-21
*MYBPC3*	2.20E-21	PTV+missPopEVE MAF_max_<0.001	3.1 [2.5; 3.9]	677	2.94E-21	8.57E-22	4.46E-02	5.10E-17
*KDM5B*	2.44E-15	PTVnf+missREVEL MAF_max_<0.01	1.9 [1.6; 2.2]	1881	1.18E-14	1.55E-11	7.58E-07	1.47E-12
*PKP2*	1.79E-14	PTV+missREVEL MAF_max_<0.001	1.8 [1.5; 2.1]	2080	2.19E-14	1.29E-14	2.40E-03	8.50E-12
*ACTC1*	3.02E-09	PTV+missAM MAF_max_<0.001	3 [2.2; 4.2]	365	3.62E-09	8.16E-03	1.95E-08	1.49E-07
*ENTREP1*	1.36E-08	PTVnf+missPAI3D MAF_max_<0.01	1.6 [1.4; 1.9]	2207	2.51E-08	2.14E-04	2.62E-06	1.03E-08
*PLEC*	1.40E-08	PTV+miss3/4 MAF_max_<1e-05	2.3 [1.8; 2.9]	618	9.32E-09	6.99E-09	5.05E-04	5.90E-10
*RBM20*	6.70E-08	PTVnf+miss3/4 MAF_max_<0.001	2 [1.6; 2.5]	738	8.87E-08	5.86E-05	7.82E-05	1.10E-06
*CTNNA3*	7.01E-08	PTV+miss3/4 MAF_max_<0.001	1.7 [1.4; 2.1]	1160	9.49E-08	3.74E-08	1.26E-02	1.97E-07
*SCN5A*	2.08E-07	missAM MAF_max_<0.01	0.8 [0.7; 0.9]	9762	8.34E-06	1.11E-02	7.02E-08	5.43E-08
*FNIP1*	4.12E-07	PTV+missPopEVE MAF_max_<0.001	3.8 [2.3; 6.1]	152	5.47E-07	1.61E-07	5.68E-01	2.24E-04
*ZFP36L2*	7.96E-07	PTV+miss3/4 MAF_max_<0.001	4.4 [2.6; 7.4]	131	1.04E-06	9.38E-07	4.68E-01	1.79E-04
*FBN1*	2.49E-06	PTV+miss3/4 MAF_max_<1e-05	1.9 [1.5; 2.4]	815	1.66E-06	2.87E-05	3.90E-04	6.54E-05

Note: OR, odds ratio; cMAC, cumulative minor allele count; MAFmax, population-maximum allele frequency; PTV, protein-truncating variants; EUR, European ancestry; miss3/4, missense variants with at least ¾ bioinformatic tools predicting a damaging effect; miss1/4, missense variants with at least ¼ bioinformatic tools predicting a damaging effect; PTVnf; proteintruncating variants without any flag; missREVEL, missense variants predicted as damaging by the REVEL bioinformatic tool; missAM, missense variants predicted as damaging by the AlphaMissense tool; missPAI3D, missense variants predicted as damaging by the PrimateAI-3D tool; missPopEVE, missense variants predicted as damaging by the PopEVE tool.

## Data Availability

Summary results for the main analyses will be made available through the Cardiovascular Disease Knowledge Portal (https://cvd.hugeamp.org) upon publication. Access to individual level UK Biobank data, both phenotypic and genetic, is available to approved researchers through application on the UK Biobank website (https://www.ukbiobank.ac.uk). The UK Biobank exome sequencing data can be found in the UK Biobank showcase portal https://biobank.ndph.ox.ac.uk/showcase/label.cgi?id=170. Additional information about registration for access to the data is available at http://www.ukbiobank.ac.uk/register-apply/. Use of UK Biobank data was performed under applications 17488 and 176602. TOPMed genomic data and pre-existing Parent study phenotypic data are made available to the scientific community in study-specific accessions in the database of Genotypes and Phenotypes (dbGaP; https://www.ncbi.nlm.nih.gov/gap/advanced_search/?TERM=topmed). Individual-level data (including raw sequencing data) for several of the CCDG-WES sub-cohorts have been deposited to dbGAP/AnVIL under restricted access (ENGAGE-TIMI: phs002774; PEGASUS-TIMI: phs002243; MGB: phs002018; MGH_AF: phs001062; TMDU: phs002985; BioVu: phs001624; SWISS_AF: phs002242; DECAF/TCAI: phs001546; VAFAR: phs000997; UCSF_AF: phs001933; GENAF: phs001547; GGAF: phs001725). Access to individual phenotypic and genetic data from All of Us Research Program is available to bona fide researchers with institutional data use agreements, through the Researcher Workbench, a cloud-based computing platform (https://www.researchallofus.org/register/). A publicly available data browser is provided by the research program (https://databrowser.researchallofus.org/). Access to Geisinger MyCode individual-level data is restricted but available through data access agreements with Regeneron https://regeneron.envisionpharma.com/vt_regeneron/. Individual-level data for MGB and the various TIMI clinical trials are not publicly available at this time due to the sensitive nature of the data and associated consents. Data from the National Genomic Research Library (NGRL) used in this research are available within the secure Genomics England Research Environment. Access to NGRL data is restricted to adhere to consent requirements and protect participant privacy. Data used in this research include the AggV2 WGS variant call sets, associated annotation data, and diagnosis billing code data; access to NGRL data is provided to approved researchers who are members of the Genomics England Research Network, subject to institutional access agreements and research project approval under participant-led governance (https://www.genomicsengland.co.uk/research). Other datasets used in this manuscript include: the Human Genome assembly GRCh38 (https://www.ncbi.nlm.nih.gov/datasets/genome/GCF_000001405.26/); the dbNSFP database v.4.2 and v4.3; (https://sites.google.com/site/jpopgen/dbNSFP); gnomAD exomes v.2.1 (https://gnomad.broadinstitute.org/downloads); Ensembl release 104 and 105 (https://www.ensembl.org/info/data/index.html); popEVE, AlphaMissense, and PrimateAI-3D variant predictions (all from the initial publication releases).

## Code Availability

For most datasets, data collection and data pre-processing were performed centrally, and therefore no commercial software was needed to collect data specific to the present study. Pre-processing of sequencing data for the CCDG-WES, MGB, FOURIER and DECLARE datasets were performed using various versions of the Genome Analysis Toolkit (https://github.com/broadinstitute/gatk/releases), as described in the **Supplementary Note**. Quality-control of individual-level data was performed using Hail version 0.2 (https://hail.is), PLINK version 2.0.a (https://www.cog-genomics.org/plink/2.0/), and KING version 2.2.5 (https://www.kingrelatedness.com/Download.shtml), as specified in the **Supplementary Note**. Variant annotation was performed using VEP versions 104 and 105 (https://github.com/Ensembl/ensembl-vep) including the LOFTEE plug-in (https://github.com/Ensembl/ensembl-vep) run within Hail. Association tests were performed using REGENIE (versions 3 and 4). Meta-analyses were performed using METAL (2017–12-21 release), and post-processed using a custom script to perform a count-based Saddle-point-approximation correction (available in the GitHub repository https://github.com/seanjosephjurgens/UKBB_200KWES_CVD/v.1.2). Analyses that were run in R, were run within R v.4 (https://www.r-project.org).

## Consortium authors and affiliations

### Regeneron Genetics Center

#### * Authors are shown in alphabetical order by surname

Gonçalo Abecasis_84,90_, Xiaodong Bai_84_, Suganthi Balasubramanian_84_, Aris Baras_84_, Christina Beechert_84_, Boris Boutkov_84_, Michael Cantor_84_, Giovanni Coppola_84_, Tanima De_84_, Andrew Deubler_84_, Aris Economides_84_, Gisu Eom_84_, Manuel AR Ferreira_84_, Caitlin Forsythe_84_, Erin D Fuller_84_, Zhenhua Gu_84_, Lukas Habegger_84_, Alicia Hawes_84_, Marcus B Jones_84_, Katia Karalis_84_, Shareef Khalid_84_, Olga Krasheninina_84_, Rouel Lanche_84_, Michael Lattari_84_, Dadong Li_84_, Alexander Lopez_84_, Luca A Lotta_84_, Kia Manoochehri_84_, Adam J Mansfield_84_, Evan K Maxwell_84_, Jason Mighty_84_, Lyndon J Mitnaul_84_, Mona Nafde_84_, Jonas Nielsen_84_, Sean O’Keeffe_84_, Max Orelus_84_, John D Overton_84_, Maria Sotiropoulos Padilla_84_, Razvan Panea_84_, Tommy Polanco_84_, Manasi Pradhan_84_, Ayesha Rasool_84_, Jeffrey G Reid_84_, William Salerno_84_, Thomas D Schleicher_84_, Alan Shuldiner_84_, Katherine Siminovitch_84_, Jeffrey C Staples_84_, Ricardo H Ulloa_84_, Niek Verweij_84_, Louis Widom_84_, Sarah E Wolf_84_

#### NHLBI Trans-Omics for Precision Medicine (TOPMed) Consortium

* Authors are shown in order as presented in the main author list, if present in main author list; all remaining TOPMed authors are presented after the main authors in alphabetical order by surname

Seung Hoan Choi_4_, Lu-Chen Weng_1,8_, Mark D Chaffin_1_, Alanna C Morrison_21_, Brian Cade_26_, Jennifer A Brody_34_, John Barnard_35_, John E Hokanson_36_, Joshua C Bis_34_, Leann Long_38_, Michael Preuss_44_, Namrata Gupta_45_, Nicholas L Smith_47,48_, Stacey Gabriel_45_, Yuan-I Min_60_, Alvaro Alonso_62_, Bruce M Psaty_34,47_, Christine M Albert_63_, Dan E Arking_64_, Dan M Roden_52_, Daniel I Chasman_45,65_, Eric Boerwinkle_21_, Laura M Raffield_73_, M. Benjamin Shoemaker_30_, Michael H Cho_74_, Mina K Chung_75_, Nona Sotoodehnia_34_, Ruth JF Loos_44,78_, Susan R Heckbert_48,81_, Susan Redline_26_, Dawood Darbar_15_, Steven A Lubitz_86_, Kathryn L Lunetta_4,87_, Emelia J Benjamin_88,87_, Patrick T Ellinor_1,8,86_, Gonçalo Abecasis_84,90_, Francois Aguet_91_, Laura Almasy_92,93_, Seth Ament_94_, Peter Anderson_95_, Pramod Anugu_96_, Kristin Ardlie_91_, Donna K Arnett_97_, Allison Ashley-Koch_98_, Stella Aslibekyan_99_, Tim Assimes_100_, Paul Auer_101_, Dimitrios Avramopoulos_102_, Najib Ayas_103_, Kathleen Barnes_104,105_, R. Graham Barr_106_, Emily Barron-Casella_102_, Terri Beaty_102_, Gerald Beck_107_, Diane Becker_102_, Lewis Becker_102_, Amber Beitelshees_94_, Marcos Bezerra_108_, Larry Bielak_90_, John Blangero_109_, Nathan Blue_110_, Donald W. Bowden_111_, Russell Bowler_107_, Ulrich Broeckel_101_, Deborah Brown_112_, Esteban Burchard_113_, Carlos Bustamante_100_, Jonathan Cardwell_114_, Vincent Carey_115_, April P. Carson_96_, Cara Carty_116_, Richard Casaburi_117_, James Casella_102_, Peter Castaldi_115_, Christy Chang_94_, Yi-Cheng Chang_118_, Sameer Chavan_114_, Wei-Min Chen_119_, Yii-Der Ida Chen_120_, Lee-Ming Chuang_118_, Ren-Hua Chung_121_, Clary Clish_91_, Suzy Comhair_107_, Matthew Conomos_95_, Elaine Cornell_122_, Carolyn Crandall_117_, James Crapo_123_, L. Adrienne Cupples_124_, Joanne Curran_109_, Jeffrey Curtis_90_, Brian Custer_125_, Coleen Damcott_94_, Sean David_126_, Colleen Davis_95_, Michelle Daya_114_, Mariza de Andrade_127_, Lisa de las Fuentes_128_, Paul de Vries_112_, Michael DeBaun_129_, Ranjan Deka_130_, Dawn DeMeo_115_, Scott Devine_94_, Harsha Doddapaneni_131_, Qing Duan_132_, Shannon Dugan-Perez_131_, Ravi Duggirala_109_, Jon Peter Durda_122_, Susan K. Dutcher_128_, Charles Eaton_133_, Lynette Ekunwe_96_, Adel El Boueiz_134_, Serpil Erzurum_107_, Charles Farber_119_, Tasha Fingerlin_123_, Myriam Fornage_112_, Nora Franceschini_132_, Chris Frazar_95_, Mao Fu_94_, Stephanie M. Fullerton_95_, Lucinda Fulton_128_, Weiniu Gan_135,136_, Shanshan Gao_114_, Margery Gass_137_, Heather Geiger_138_, Bruce Gelb_139_, Mark Geraci_140_, Soren Germer_138_, Robert Gerszten_141_, Auyon Ghosh_115_, Richard Gibbs_131_, Mark Gladwin_140_, David Glahn_142,143_, Stephanie Gogarten_95_, Harald Goring_109_, Sharon Graw_105_, Kathryn J. Gray_95_, C. Charles Gu_128_, Xiuqing Guo_120_, Jeff Haessler_137_, Michael Hall_96_, Patrick Hanly_144_, Daniel Harris_94_, Nicola L. Hawley_145_, Jiang He_146_, Ben Heavner_95_, Ryan Hernandez_113_, David Herrington_111_, Craig Hersh_115_, Bertha Hidalgo_99_, James Hixson_112_, Brian Hobbs_115_, Karin Hoth_147_, Chao (Agnes) Hsiung_121_, Chii Min Hwu_148_, Marguerite Ryan Irvin_99_, Cashell Jaquish_135,136_, Jill Johnsen_95_, Andrew Johnson_135,136_, Craig Johnson_95_, Rich Johnston_149_, Kimberly Jones_102_, Robert Kaplan_150_, Sharon Kardia_90_, Shannon Kelly_113_, Eimear Kenny_139_, Michael Kessler_94_, Alyna Khan_95_, Wonji Kim_134_, Greg Kinney_114_, Barbara Konkle_151_, Charles Kooperberg_137_, Holly Kramer_152_, Christoph Lange_153_, Ethan Lange_114_, Cathy Laurie_95_, Meryl LeBoff_115_, Wen-Jane Lee_148_, Jonathon LeFaive_90_, Dan Levy_135,136_, Joshua Lewis_94_, Xiaohui Li_120_, Yun Li_132_, Henry Lin_120_, Honghuang Lin_124_, Xihong Lin_153_, Simin Liu_154_, Yongmei Liu_98_, Yu Liu_100_, James Luo_135,136_, Ulysses Magalang_155_, Michael Mahaney_109_, Barry Make_102_, Ani Manichaikul_119_, Alisa Manning_91,134,156_, JoAnn Manson_115_, Lisa Martin_157_, Melissa Marton_138_, Susan Mathai_114_, Rasika Mathias_136_, Patrick McArdle_94_, Merry-Lynn McDonald_99_, Becky McNeil_158_, Hao Mei_96_, James Meigs_156_, Luisa Mestroni_105_, Ginger Metcalf_131_, Deborah A Meyers_127_, Emmanuel Mignot_100_, Julie Mikulla_135,136_, Ryan L Minster_140_, Braxton D. Mitchell_94_, Matt Moll_115_, May Montasser_135_, Courtney Montgomery_159_, Donna Muzny_131_, Josyf C Mychaleckyj_119_, Girish Nadkarni_139_, Rakhi Naik_102_, Pradeep Natarajan_91_, Sergei Nekhai_160_, Sarah C. Nelson_95_, Kari North_132_, Jeff O’Connell_94_, Tim O’Connor_94_, Heather Ochs-Balcom_161_, Allan Pack_93_, Nicholette Palmer_111_, James Pankow_162_, Gina Peloso_124_, Juan Manuel Peralta_109_, Marco Perez_100_, James Perry_94_, Ulrike Peters_137_, Patricia Peyser_90_, Lawrence S Phillips_149_, Toni Pollin_94_, Wendy Post_102_, Julia Powers Becker_114_, Meher Preethi Boorgula_114_, Pankaj Qasba_135_, Dandi Qiao_115_, Zhaohui Qin_149_, Nicholas Rafaels_114_, D.C. Rao_128_, Laura Rasmussen-Torvik_163_, Aakrosh Ratan_119_, Robert Reed_94_, Catherine Reeves_138_, Elizabeth Regan_123_, Alex Reiner_137,95_, Rebecca Robillard_164_, Nicolas Robine_138_, Carolina Roselli_91_, Jerome Rotter_120_, Ingo Ruczinski_102_, Alexi Runnels_138_, Sarah Ruuska_151_, Kathleen Ryan_94_, Ester Cerdeira Sabino_165_, Danish Saleheen_106_, Shabnam Salimi_94_, Steven Salzberg_102_, Kevin Sandow_120_, Vijay G. Sankaran_134,142_, Richa Saxena_91,166,156_, Karen Schwander_128_, David Schwartz_114_, Frank Sciurba_140_, Christine Seidman_143_, Jonathan Seidman_143_, Vivien Sheehan_149_, Stephanie L. Sherman_149_, Amol Shetty_94_, Wayne Hui-Heng Sheu_148_, Brian Silver_167_, Albert Vernon Smith_90_, Jennifer Smith_90_, Josh Smith_95_, Sylvia Smoller_150_, Beverly Snively_111_, Michael Snyder_100_, Tamar Sofer_141_, Adrienne M. Stilp_95_, Garrett Storm_114_, Elizabeth Streeten_94_, Jessica Lasky Su_115_, Yun Ju Sung_128_, Adam Szpiro_95_, Hua Tang_100_, Margaret Taub_102_, Kent D. Taylor_120_, Matthew Taylor_105_, Simeon Taylor_94_, Marilyn Telen_98_, Timothy A. Thornton_95_, Machiko Threlkeld_95_, Lesley Tinker_137_, David Tirschwell_95_, Sarah Tishkoff_93_, Hemant Tiwari_99_, Catherine Tong_95_, Russell Tracy_122_, Michael Tsai_162_, Dhananjay Vaidya_102_, David Van Den Berg_168_, Scott Vrieze_162_, Tarik Walker_114_, Robert Wallace_147_, Heming Wang_115,166_, Jiongming Wang_90_, Karol Watson_117_, Daniel E. Weeks_140_, Joshua Weinstock_149_, Jennifer Wessel_169_, L. Keoki Williams_170_, Scott Williams_171_, Carla Wilson_115_, Lara Winterkorn_138_, Baojun Wu_170_, Joseph Wu_100_, Huichun Xu_94_, Lisa Yanek_102_, Ivana Yang_114_, Ronit Yarden_136,135_, Seyedeh Maryam Zekavat_91_, Yingze Zhang_140_, Wei Zhao_90_, Xiaofeng Zhu_171_, Elad Ziv_113_, Michael Zody_138_

#### Additional affiliations

_90_University of Michigan, Ann Arbor, MI, USA, _91_Broad Institute, Cambridge, MA, USA, _92_Children’s Hospital of Philadelphia, Philadelphia, PA, USA, _93_University of Pennsylvania, Philadelphia, PA, USA, _94_University of Maryland, College Park, MD, USA, _95_University of Washington, Seattle, WA, USA, _96_University of Mississippi, Oxford, MS, USA, _97_University of South Carolina, Columbia, SC, USA, _98_Duke University, Durham, NC, USA, _99_University of Alabama, Tuscaloosa, AL, USA, _100_Stanford University, Stanford, CA, USA, _101_Medical College of Wisconsin, Milwaukee, WI, USA, _102_Johns Hopkins University, Baltimore, MD, USA, _103_Providence Health Care, Vancouver, BC, Canada, _104_Tempus, Chicago, IL, USA, _105_University of Colorado Anschutz Medical Campus, Aurora, CO, USA, _106_Columbia University, New York, NY, USA, _107_Cleveland Clinic, Cleveland, OH, USA, _108_Fundação de Hematologia e Hemoterapia de Pernambuco - Hemope, Recife, Pernambuco, Brazil, _109_University of Texas Rio Grande Valley School of Medicine, Edinburg, TX, USA, _110_University of Utah, Salt Lake City, UT, USA, _111_Wake Forest Baptist Health, Winston-Salem, NC, USA, _112_University of Texas Health at Houston, Houston, TX, USA, _113_University of California San Francisco, San Francisco, CA, USA, _114_University of Colorado at Denver, Denver, CO, USA, _115_Brigham & Women’s Hospital, Boston, MA, USA, _116_Washington State University, Pullman, WA, USA, _117_University of California Los Angeles, Los Angeles, CA, USA, _118_National Taiwan University, Taipei, Taiwan, _119_University of Virginia, Charlottesville, VA, USA, _120_Lundquist Institute, Torrance, CA, USA, _121_National Health Research Institute Taiwan, Zhunan, Miaoli, Taiwan, _122_University of Vermont, Burlington, VT, USA, _123_National Jewish Health, Denver, CO, USA, _124_Boston University, Boston, MA, USA, _125_Vitalant Research Institute, San Francisco, CA, USA, _126_University of Chicago, Chicago, IL, USA, _127_Mayo Clinic, Rochester, MN, USA, _128_Washington University in St Louis, St. Louis, MO, USA, _129_Vanderbilt University, Nashville, TN, USA, _130_University of Cincinnati, Cincinnati, OH, USA, _131_Baylor College of Medicine Human Genome Sequencing Center, Houston, TX, USA, _132_University of North Carolina, Chapel Hill, NC, USA, _133_Brown University, Providence, RI, USA, _134_Harvard University, Cambridge, MA, USA, _135_National Heart, Lung, and Blood Institute, Bethesda, MD, USA, _136_National Institutes of Health, Bethesda, MD, USA, _137_Fred Hutchinson Cancer Research Center, Seattle, WA, USA, _138_New York Genome Center, New York, NY, USA, _139_Icahn School of Medicine at Mount Sinai, New York, NY, USA, _140_University of Pittsburgh, Pittsburgh, PA, USA, _141_Beth Israel Deaconess Medical Center, Boston, MA, USA, _142_Boston Children’s Hospital, Boston, MA, USA, _143_Harvard Medical School, Boston, MA, USA, _144_University of Calgary, Calgary, AB, Canada, _145_Yale University, New Haven, CT, USA, _146_Tulane University, New Orleans, LA, USA, _147_University of Iowa, Iowa City, IA, USA, _148_Taichung Veterans General Hospital Taiwan, Taichung, Taiwan, _149_Emory University, Atlanta, GA, USA, _150_Albert Einstein College of Medicine, Bronx, NY, USA, _151_Blood Works Northwest, Seattle, WA, USA, _152_Loyola University, Chicago, IL, USA, _153_Harvard School of Public Health, Boston, MA, USA, _154_University of California Irvine, Irvine, CA, USA, _155_The Ohio State University, Columbus, OH, USA, _156_Massachusetts General Hospital, Boston, MA, USA, _157_George Washington University, Washington, DC, USA, _158_RTI International, Research Triangle Park, NC, USA, _159_Oklahoma Medical Research Foundation, Oklahoma City, OK, USA, _160_Howard University, Washington, DC, USA, _161_University at Buffalo, Buffalo, NY, USA, _162_University of Minnesota, Minneapolis, MN, USA, _163_Northwestern University, Evanston, IL, USA, _164_University of Ottawa, Ottawa, ON, Canada, _165_Universidade de Sao Paulo, São Paulo, SP, Brazil, _166_Mass General Brigham, Boston, MA, USA, _167_UMass Memorial Medical Center, Worcester, MA, USA, _168_University of Southern California, Los Angeles, CA, USA, _169_Indiana University, Bloomington, IN, USA, _170_Henry Ford Health System, Detroit, MI, USA, _171_Case Western Reserve University, Cleveland, OH, USA
